# Fooled by His Imagination

**Published:** 1896-10

**Authors:** 


### Fooled by His Imagination.

The following story comes from France. Two travelers met in a hotel. There was but one vacant room for both, and there was no alternative but to share this room and its one bed.

In the middle of the night, one of them wakened in great distress, crying out, "Air, air! I am smothering!"

His companion arose and hunted for the matches, but he could find none. The room was so absolutely dark that he could not find the window. The sick man continued to cry "Air! I am smothering! Break the glass if you can not open the window!" So a pane was smashed.

His asthmatic companion was now relieved, and after expressing thanks, went to sleep.

In the morning they found the windows intact; they had only broken the glass of a bookcase. This satisfied the mind of the restless sleeper. It was to him sufficient. So it often is in other matters. We imagine many of our troubles. The light of knowledge would show they were only phantoms, creations of our own imagination.—*Journal of Hygiene.*

